# Mechanotransduction in glioma stem cell fate determination: from niche mechanics to therapeutic vulnerability and state plasticity

**DOI:** 10.3389/fcell.2026.1849535

**Published:** 2026-06-12

**Authors:** Yong Ai, Jiayu Zhou

**Affiliations:** 1 Department of Pathology, Hunan Maternal and Child Health Hospital, Changsha, China; 2 School of Clinical Medicine, Tsinghua University, Beijing, China

**Keywords:** extracellular matrix stiffness, glioblastoma, glioma stem cells, mechanical memory, mechanotransduction, Piezo1, tumor microenvironment, YAP/TAZ

## Abstract

Glioblastoma (GBM), the most aggressive primary brain malignancy, harbors glioma stem cells (GSCs) that drive tumor initiation, therapeutic resistance, and recurrence. While the biochemical regulation of GSCs through Notch, Wnt, and Hedgehog pathways is well established, accumulating evidence reveals that the physical and mechanical properties of the tumor microenvironment—including matrix stiffness, solid stress, viscoelasticity, and topographical architecture—constitute a fundamental yet underappreciated layer of GSC regulation. This review provides a comprehensive synthesis of how mechanotransduction pathways regulate GSC fate decisions, including self-renewal, differentiation, migration, and drug resistance. We systematically characterize the mechanical properties of distinct GSC niches, delineate the key mechanosensors and signaling cascades—including integrins/FAK, CD44-hyaluronic acid, Piezo1 channels, and YAP/TAZ-Hippo signaling—that connect extracellular mechanics to intracellular fate decisions. Crucially, aligning with recent single-cell transcriptomic paradigms, we conceptualize GSCs not as a static cellular hierarchy, but as highly plastic populations transitioning among dynamic states (e.g., neural progenitor-like, mesenchymal-like). We examine how mechanical cues serve as profound biophysical drivers of this state plasticity, alongside the emerging roles of nuclear mechanotransduction and mechanical memory. We critically evaluate the discrepancy between 2D and 3D mechanical responses, consolidate the ongoing debate regarding GBM tissue stiffness, and assess the therapeutic potential of targeting mechanotransduction to eliminate GSCs. By integrating insights from mechanobiology, stem cell biology, neuro-oncology, and bioengineering, we propose the “mechano-stemness axis” as a conceptual framework positioning mechanical forces as critical modulators of GSC dynamic plasticity, and identify critical knowledge gaps requiring direct validation in patient-derived models.

## 1 Introduction

GBM, classified as a WHO grade IV glioma, remains the most aggressive and lethal primary malignant tumor of the central nervous system, with a median survival of approximately 14–16 months despite multimodal treatment combining maximal surgical resection, radiotherapy, and temozolomide chemotherapy (Stupp et al., 2005). A defining hallmark of GBM is its remarkable intratumoral heterogeneity, which is increasingly attributed to a subpopulation of self-renewing, tumorigenic cells termed GSCs. Since their initial prospective identification by Singh et al. (2004). GSCs have been established as central drivers of tumor initiation, therapeutic resistance, and inevitable recurrence. GSCs reside in specialized anatomical niches—including perivascular, hypoxic, and invasive front regions—where biochemical signals such as Notch, Wnt, Hedgehog, and growth factor pathways cooperate to maintain stemness and regulate differentiation (Lathia et al., 2015). However, accumulating evidence over the past decade has revealed that the regulation of GSC fate extends far beyond soluble molecular cues, encompassing the physical and mechanical properties of the tumor microenvironment.

The brain, once considered a mechanically inert tissue, is now recognized as a highly mechanosensitive organ in which tissue stiffness, viscoelasticity, and topographical architecture profoundly influence cellular behavior. Normal brain parenchyma is among the softest tissues in the body, with an elastic modulus typically ranging from 0.1 to 1kPa (Budday et al., 2015). Glioma progression substantially alters this mechanical landscape. Tumor growth generates compressive solid stress that can reach several kilopascals, capable of collapsing blood vessels and distorting surrounding parenchyma (Nia et al., 2016). Concurrently, extensive remodeling of the extracellular matrix (ECM)—including aberrant deposition of hyaluronic acid (HA), tenascin-C, and fibronectin—creates regions of increased stiffness and altered viscoelasticity within and around the tumor (Miroshnikova et al., 2016). These mechanical changes are not merely passive consequences of tumor expansion; rather, they actively instruct cell fate decisions through mechanotransduction—the process by which cells convert mechanical stimuli into biochemical signals.

A rapidly expanding body of literature has demonstrated that mechanical cues from the extracellular environment are transduced into intracellular signaling cascades through an array of mechanosensors and mechanotransducers. Integrins, the principal transmembrane receptors for ECM components, form focal adhesion complexes that activate focal adhesion kinase (FAK) and downstream Rho-GTPase signaling, directly coupling matrix rigidity to cytoskeletal remodeling and gene expression (Pang et al., 2017). The mechanosensitive ion channels Piezo1 and Piezo2 have emerged as critical mediators in glioma biology; Chen et al. (2018) demonstrated that Piezo1 activation by tissue stiffness in glioma cells engages an integrin-FAK-dependent feedforward loop that amplifies tumor aggressiveness. Perhaps most critically, the transcriptional co-activators YAP (Yes-associated protein) and TAZ (transcriptional co-activator with PDZ-binding motif), the core effectors of the Hippo signaling pathway, have been identified as master integrators of mechanical inputs in glioma. Nuclear translocation of YAP/TAZ in response to increased ECM stiffness drives transcriptional programs that promote proliferation, survival, and mesenchymal transition in GBM cells (Totaro et al., 2018). The downstream consequences of these cytoplasmic mechanotransduction cascades ultimately converge on the nucleus. Extracellular mechanical signals, once transduced into cytoskeletal tension, are physically coupled to the nucleus via the LINC (Linker of Nucleoskeleton and Cytoskeleton) complex. This physical bridge connects the cytoskeleton to the nuclear lamina and transmits mechanical forces directly to the chromatin, where they can alter chromatin organization and epigenetic states, thereby influencing gene expression programs associated with GSC identity (Guilluy et al., 2014).

Despite these significant individual discoveries, a critical gap remains in our understanding of how biophysical forces regulate GSC identity. Crucially, the conceptual framework of GSCs has recently undergone a paradigm shift. Landmark single-cell transcriptomic studies have redefined GSCs from a static, hierarchical cell population into highly plastic and dynamic entities capable of transitioning among distinct cellular states (e.g., oligodendrocyte progenitor-like [OPC-like], astrocyte-like, and mesenchymal-like) in response to microenvironmental stress (Neftel et al., 2019; Hara et al., 2021). While recent excellent reviews by Bhargav et al. (2021) and Gomes et al. (2025) have comprehensively summarized the broad impact of mechanotransduction on general brain tumor microenvironments and theranostics, the specific biophysical mechanisms governing dynamic GSC state plasticity remain largely unexplored. Therefore, this review aims to bridge this critical knowledge gap. Several lines of evidence underscore the importance and timeliness of addressing this gap. First, elegant studies using tunable hydrogel systems have shown that matrix stiffness alone can determine whether patient-derived GSCs maintain stemness or undergo differentiation. Kondapaneni et al. (2024) recently emphasized that softer substrates mimicking normal brain tissue tend to support GSC self-renewal, while stiffer matrices reflective of the tumor bulk can promote proliferative or mesenchymal phenotypes, suggesting that the mechanical heterogeneity of the niche directly controls the stem cell hierarchy. Second, emerging research has revealed that the mechanical properties of distinct GSC niches differ significantly—perivascular niches are characterized by relatively higher stiffness due to the presence of the vascular basement membrane, whereas perinecrotic niches are softer and more compliant (Grundy et al., 2016)—implying that niche mechanics may underlie the phenotypic diversity of GSCs observed across different tumor compartments. Third, recent single-cell and spatial transcriptomic analyses have identified mechanosensitive gene signatures enriched in GSC populations, further linking mechanical sensing to the molecular identity of these cells (Neftel et al., 2019). Fourth, nuclear mechanobiology—a burgeoning field investigating how forces transmitted to the nucleus reshape chromatin architecture and epigenetic landscapes—has opened entirely new avenues for understanding how physical forces may regulate the epigenetic programs that maintain GSC identity (Kalukula et al., 2022). These converging lines of evidence strongly suggest that mechanotransduction represents a fundamental, yet underappreciated, layer of GSC regulation that cannot be captured by molecular analyses alone.

Furthermore, the clinical significance of understanding mechanoregulation in GSCs is substantial. The inevitable recurrence of GBM is largely attributed to treatment-resistant GSCs that survive standard therapy and repopulate the tumor. If specific mechanotransduction pathways are required for the maintenance of GSC stemness and therapy resistance, these pathways could represent novel therapeutic vulnerabilities. Importantly, unlike genetic mutations, the mechanical microenvironment is, in principle, pharmacologically and physically modifiable—for example, through ECM-targeting strategies, mechanosensitive channel inhibitors (such as GsMTx4 for Piezo channels), YAP/TAZ pathway modulators (such as verteporfin), or physical interventions including tumor treating fields (TTFields), which have already been approved for GBM treatment and exert their effects at least partially through mechanical disruption of mitotic processes (Stupp et al., 2017). The convergence of mechanobiology with precision oncology thus holds the promise of identifying mechano-based biomarkers for GSC-rich tumors and developing strategies that target the physical dimensions of the stem cell niche.

This review aims to provide a comprehensive and integrative synthesis of current knowledge on how mechanotransduction pathways regulate GSC fate determination. Specifically, the objectives of this review are: (1) to systematically characterize the mechanical properties of distinct GSC niches within the glioma microenvironment, including stiffness, viscoelasticity, solid stress, and topographical features, and how these are measured and quantified using state-of-the-art techniques; (2) to delineate the key mechanosensors and mechanotransduction cascades—including integrins/FAK, Piezo channels, YAP/TAZ-Hippo signaling, and the LINC complex/nuclear mechanotransduction—that operate in GSCs and connect extracellular mechanics to intracellular fate decisions; (3) to examine the crosstalk between mechanical signaling and canonical stemness pathways (Notch, Wnt, Hedgehog, and growth factor receptors) in GSCs; (4) to explore the emerging role of nuclear mechanics and mechano-epigenetics in the regulation of GSC identity and plasticity; and (5) to evaluate the therapeutic potential of targeting mechanotransduction pathways to eliminate GSCs or force their differentiation, with particular attention to translational feasibility and ongoing clinical efforts. By integrating insights from mechanobiology, stem cell biology, neuro-oncology, and bioengineering, this review seeks to establish a coherent conceptual framework—what we term the “mechano-stemness axis”—that positions mechanical forces as central determinants of GSC fate, and to identify critical knowledge gaps and future research directions that could accelerate the translation of mechanobiological insights into improved outcomes for glioblastoma patients.

**FIGURE 1:**
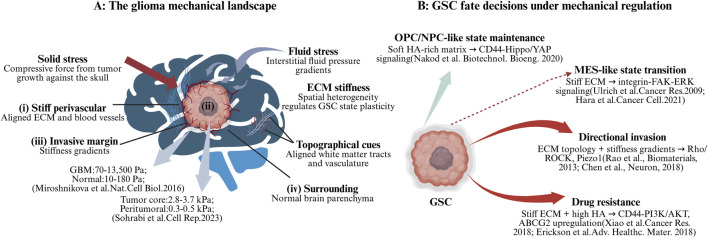
The mechanical landscape of glioblastoma and its profound influence on dynamic GSC state plasticity and fate decisions. **(A)** The macroscopic glioma mechanical landscape. A schematic cross-section illustrating the profound spatial heterogeneity of the glioblastoma (GBM) microenvironment. GSCs reside within distinct anatomical niches subjected to four major classes of biophysical cues: compressive solid stress (arising from tumor expansion against the rigid skull), interstitial fluid pressure gradients, topographical guidance (from aligned white matter tracts and blood vessels), and heterogeneous ECM stiffness. The distinct micromechanical profiles of these niches (e.g., rigid perivascular zones vs. softer peritumoral margins) serve as critical biophysical modulators of GSC plasticity. Representative stiffness values are derived from traceable AFM measurements of human biopsies and mouse xenografts. **(B)** GSC state plasticity and fate decisions under mechanical regulation. Extracellular mechanical cues converge on GSC behavior through distinct mechanosensor-signaling axes. Rather than dictating a static hierarchy, specific biophysical inputs drive dynamic transcriptomic and phenotypic adaptations: soft, hyaluronic acid (HA)-rich matrices maintain oligodendrocyte/neural progenitor-like (OPC/NPC-like) states via CD44-Hippo/YAP signaling. Conversely, severe mechanical conditions, such as stiff fibrotic ECM, are hypothesized to force a transition toward the highly aggressive mesenchymal-like (MES-like) state via integrin-FAK-ERK cascades. Furthermore, mechanical topography coupled with stiffness gradients directs single-cell invasion (Rho/ROCK, Piezo1), while the synergistic integration of stiff ECM and high HA concentration orchestrates therapeutic resistance. Note on line styles and evidence: Solid arrows represent firmly established behavioral responses supported by direct experimental evidence in patient-derived GSCs or established bulk glioma models. The dashed arrow denotes a proposed mechanism (i.e., stiffness-driven forced MES-like state transition); while strongly supported by recent multi-omics and mechanobiological correlations, this concept represents an emerging hypothesis that requires direct, causal validation in primary GSC models. Pathway and behavioral annotations are derived from the accompanying traceable references. Created in BioRender. Ai, Y. (2026) https://BioRender.com/p4sr4q8.

A schematic overview of the spatially heterogeneous mechanical landscape of GBM and the convergence of mechanical cues on GSC fate decisions through distinct mechanotransduction pathways is presented in Figure 1. As illustrated in Figure 1A, the GBM tumor microenvironment encompasses mechanically distinct compartments—including a stiff perivascular niche with aligned ECM and vascular basement membrane, a soft hypoxic/necrotic core, an invasive margin with intermediate stiffness gradients, and the surrounding normal brain parenchyma—each characterized by different combinations of solid stress, fluid stress, ECM stiffness, and topographical guidance cues. Stiffness values annotated in this schematic are derived from AFM measurements of mouse GBM xenograft tissue, which demonstrated tumor core stiffness of 2.8–3.7 kPa, peritumoral tissue stiffness of 0.3–0.5 kPa, and tumor edge stiffness of 1.2–1.3 kPa (Sohrabi et al., 2023), as well as AFM measurements of human GBM biopsies spanning 70–13,500 Pa compared with 10–180 Pa in normal tissue (Miroshnikova et al., 2016). As shown in Figure 1B, these mechanical cues converge on GSC fate decisions through pathway-specific signaling: soft HA-rich matrices promote self-renewal via CD44-Hippo/YAP signaling (Kim and Kumar, 2014; Dupont et al., 2011); stiff ECM drives differentiation through integrin-FAK-ERK cascades (Ulrich et al., 2009; Umesh et al., 2014); ECM topology combined with stiffness gradients regulates migration and invasion via Rho/ROCK and Piezo1 (Chen et al., 2018; Johnson et al., 2009); and the combination of stiff ECM and high HA concentration promotes drug resistance through CD44-PI3K/AKT-mediated upregulation of survival pathways (Xiao et al., 2018). This integrative framework—the “mechano-stemness axis”—serves as the organizational principle for the detailed mechanistic discussions that follow.

## 2 Evolution of understanding the glioma mechanical microenvironment

### 2.1 From biochemical-centric to mechano-biological paradigms

For decades, glioma research was dominated by a biochemical paradigm focused on genetic mutations, growth factor signaling, and hypoxic responses. The recognition that mechanical forces could independently drive tumor progression emerged gradually from pioneering work in breast cancer mechanobiology. In 2005, Paszek et al. (2005) demonstrated that ECM stiffness alone could alter integrin subtype expression, enhance focal adhesion assembly, and disrupt mammary epithelial acinar architecture through Rho- and ERK-dependent contractility (Paszek et al., 2005). This study established the foundational concept that mechanical reciprocity between cells and their ECM constitutes a fundamental regulatory axis in cancer biology.

The conceptual framework was extended to brain tumors by Kumar and Weaver, who articulated the “force journey” of a tumor cell—from tissue dysplasia through invasion and metastasis—emphasizing that each stage involves profound changes in cellular mechanical phenotype and the biophysical properties of the microenvironment (Kumar et al., 2009). However, the brain presents a unique mechanical context: it is among the softest tissues in the body (∼260–490Pa), with an ECM dominated by hyaluronic acid (HA) rather than the fibrillar collagens prevalent in breast or pancreatic tissue (Levental et al., 2007). This distinction meant that findings from stiffer tissue systems could not be directly extrapolated to glioma, necessitating brain-specific mechanobiological investigation.

**FIGURE 2:**
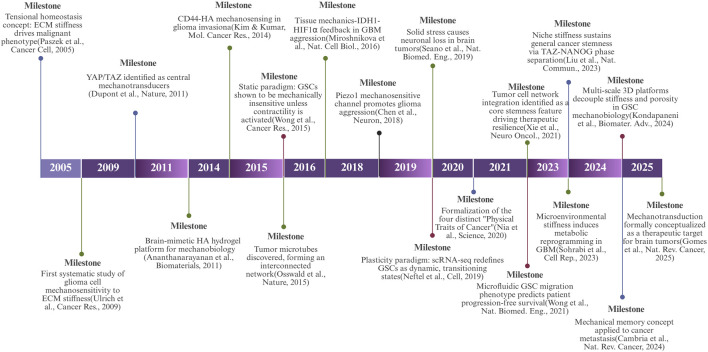
Chronological evolution of glioma mechanobiology: from foundational principles to GSC dynamic plasticity. The timeline systematically maps key milestones in the field, color-coded to rigorously delineate the scope of experimental evidence: blue nodes represent foundational concepts established in general cancer mechanobiology; green nodes denote phenomena validated in bulk glioma models or in vivo tumor biology (e.g., solid stress, tumor microtubes); and dark red nodes highlight discoveries specifically elucidating bona fide GSC behavior and advanced 3D/microfluidic platforms. Crucially, this timeline captures a profound conceptual paradigm shift in neuro-oncology. Early investigations approached mechanosensitivity through the lens of a static GSC hierarchy (e.g., Wong et al., 2015). However, the integration of in vivo imaging identifying structurally resilient tumor microtube networks capable of resisting therapeutic and physical damage (Osswald et al., 2015; Xie et al., 2021) and single-cell transcriptomics (Neftel et al., 2019) redefined GSCs as highly plastic entities capable of dynamic state transitions. Concurrently, the formal canonization of distinct “physical traits of cancer” (Nia et al., 2020) established a theoretical framework that catalyzed multiscale mechanobiological investigations. Recent milestones emphasize emerging frontiers, including mechano-metabolic reprogramming (Sohrabi et al., 2023) and multiscale biophysical decoupling (Kondapaneni et al., 2024). Emerging general paradigms --such as mechanical memory (Cambria et al., 2024) and YAP/TAZ-driven phase separation (Liu et al., 2023) --are included to illustrate cutting-edge concepts extrapolated from other solid tumors that raise critical hypotheses requiring direct validation in primary GSC models. Created in BioRender. Ai, Y. (2026) https://BioRender.com/d0wxklw.

The intellectual evolution of glioma mechanobiology—from general cancer mechanobiology principles to glioma-specific and ultimately GSC-specific investigations—is summarized chronologically in Figure 2. As this timeline illustrates, the field was catalyzed by Paszek et al.'s landmark 2005 demonstration that ECM stiffness drives malignant phenotype in breast epithelium through integrin-dependent mechanotransduction (Paszek et al., 2005), which established the foundational concept of mechanical reciprocity in cancer. The first systematic application of these principles to glioma came 4 years later with Ulrich et al.’s characterization of glioma cell mechanosensitivity (Ulrich et al., 2009), followed rapidly by the development of brain-mimetic HA hydrogel platforms (Ananthanarayanan et al., 2011) and the identification of YAP/TAZ as central mechanotransducers (Dupont et al., 2011). A critical inflection point occurred in 2015 when Wong et al. reported the paradoxical mechanical insensitivity of glioma stem cells (Wong et al., 2015), which fundamentally challenged the assumption that all glioma cells respond uniformly to mechanical cues. Subsequent discoveries—including the identification of Piezo1 as a mechanosensitive ion channel in glioma (Chen et al., 2018), the elucidation of tissue mechanics–IDH1–tenascin C feedback loops (Miroshnikova et al., 2016), and the recent recognition that stiffness induces metabolic reprogramming (Sohrabi et al., 2023) and that mechanical memory may encode persistent epigenetic changes in cancer cells (Cambria et al., 2024)—have progressively expanded the scope of glioma mechanobiology from cell-level mechanics to systems-level integration of mechanical, epigenetic, and metabolic regulation. Notably, the timeline reveals a significant temporal lag between discoveries in general cancer mechanobiology (blue milestones in Figure 2) and their translation to glioma-specific (green) and GSC-specific (red) contexts, underscoring the need for accelerated investigation of mechano-stemness mechanisms in brain tumors.

### 2.2 The stiffness controversy: is glioma stiffer or softer than normal brain?

A central and still-unresolved controversy in glioma mechanobiology concerns the relative stiffness of tumor tissue versus normal brain. This debate has significant implications: if tumors are stiffer, then stiffness-driven mechanotransduction pathways would be activated within the tumor; if softer, then cells migrating from tumor to stiff peritumoral tissue would encounter increasing mechanical resistance.

The debate originated from the use of different measurement techniques at different length scales. *In vivo* MRE studies consistently reported that GBM tumors are softer than surrounding brain parenchyma. Streitberger et al. measured a complex shear modulus of 1.32 ± 0.26 kPa in GBM versus 1.54 ± 0.27 kPa in healthy tissue (Streitbe et al., 2014). Pepin et al. extended this by showing a progressive decrease in stiffness with increasing glioma grade (Grade II: 2.7 ± 0.7 kPa; Grade IV: 1.7 ± 0.5 kPa) (Pepin et al., 2018). Longitudinal MRE studies in mouse xenograft models confirmed this softening over time (Schregel et al., 2018).

However, contradictory evidence emerged from techniques operating at finer spatial resolution. Using intraoperative SWE, Chauvet et al. reported that high-grade gliomas (11.4 ± 3.6 kPa) were stiffer than normal brain (7.3 ± 2.1 kPa) (Chauvet et al., 2016). AFM measurements of tissue specimens also supported the stiffening interpretation: Ciesluk et al. found that freshly excised GBM tissue was stiffer (168.8 ± 32.8 Pa) than healthy brain (61.2 ± 0.4 Pa) (Cieśluk et al., 2020). Critically, Miroshnikova et al. (2016) demonstrated using AFM on frozen biopsies that GBM samples spanned a remarkably broad stiffness range (70–13,500 Pa), far exceeding normal tissue (10–180 Pa), and established a mechanistic link between tissue stiffness and GBM aggression through an IDH1-dependent HIF1α-tenascin C feedback loop (Miroshnikova et al., 2016).

A resolution to this apparent paradox came from studies revealing marked spatial heterogeneity within tumors. Sohrabi et al. showed using AFM on mouse GBM xenograft tissue slices that tumor core stiffness (2.8–3.7 kPa) was significantly higher than peritumoral tissue (0.3–0.5 kPa) and tumor edge (1.2–1.3 kPa) (Sohrabi et al., 2023). This heterogeneity suggests that MRE, which measures tissue stiffness at the millimeter scale and thus averages over these distinct regions, may underestimate the stiffness experienced by cells in specific tumor compartments, whereas AFM captures the micromechanical landscape directly relevant to cell-ECM interactions.

The quantitative data underlying this controversy, organized by measurement technique, sample type, and species, are consolidated in Table 1. This systematic comparison reveals that the apparent discrepancy between MRE and AFM measurements is largely attributable to differences in spatial resolution (millimeters versus micrometers), sample preparation (*in vivo* versus *ex vivo*), and the physical quantity measured (shear modulus versus Young’s modulus). As Table 1 demonstrates, when measurements are performed at comparable spatial scales and on comparable tissue preparations, a consistent picture emerges: GBM tumors exhibit marked intratumoral mechanical heterogeneity, with some compartments significantly stiffer and others softer than normal brain parenchyma.

**TABLE 1:** Mechanical properties of GBM tumors measured by different techniques.

Measurement technique	Measurement scale	Sample status	Species	Quantity measured	Normal brain	GBM tumor	Key finding	References
MRE	Macro-scale	*In vivo* (patients)	Human	Complex shear modulus (G*)	1.54 ± 0.27 kPa	1.32 ± 0.26 kPa	GBM softer than healthy tissue	Streitbe et al. (2014)
MRE	Macro-scale	*In vivo* (patients)	Human	Complex shear modulus (G*)	3.3 ± 0.7 kPa (white matter)	Grade IV: 1.7 ± 0.5 kPa	Stiffness decreases with grade; IDH1-mutant stiffer than wild-type	Pepin et al. (2018)
MRE	Macro-scale	*In vivo* (patients)	Human	Complex shear modulus (G*)	1.81 ± 0.23 kPa	1.10 ± 0.29 kPa	High water content and GAG accumulation soften GBM	Streitber et al. (2020)
MRE	Macro-scale	*In vivo* (mouse)	Mouse	Viscoelastic modulus	10.07 ± 0.63 kPa	Week 4: 6.24 ± 0.04 kPa	Tumor softens over time	Schregel et al. (2018)
MRE	Macro-scale	*In vivo* (mouse)	Mouse	Elastic modulus (Gd)	5.89 ± 0.21 kPa	4.80 ± 0.21 kPa	All tumor types softer than healthy tissue	Jamin et al. (2015)

Measurement technique	Measurement scale	Sample type	Species	Quantity measured	Normal brain	GBM tumor	Key finding	References
SWE	Macro/Meso-scale	*In vivo* (Intraoperative)	Human	Young’s modulus	7.3 ± 2.1 kPa	High-grade: 11.4 ± 3.6 kPa	GBM stiffer than normal; opposite to MRE findings	Chauvet et al. (2016)
AFM	Micro/Nano-scale	*Ex vivo* (Fresh tissue)	Human	Young’s modulus	61.2 ± 0.4 Pa	168.8 ± 32.8 Pa	GBM stiffer than healthy tissue	Cieśluk et al. (2020)
AFM	Micro/Nano-scale	*Ex vivo* (Frozen biopsies)	Human	Median Young’s modulus	10–180 Pa	70–13,500 Pa	Broad stiffness range in GBM; IDH1-mutant softer	Miroshnikova et al. (2016)
AFM	Micro/Nano-scale	*Ex vivo* (Fresh slices)	Mouse	Median Young’s modulus	Peritumoral: 0.3–0.5 kPa	Core: 2.8–3.7 kPa	Significant spatial heterogeneity within tumors	Sohrabi et al. (2023)

Data compiled from studies measuring GBM tumor mechanical properties using different techniques. The apparent contradiction between MRE (tumors softer) and AFM/SWE (tumors stiffer) likely reflects differences in measurement scale, frequency, and spatial averaging. MRE measures at millimeter resolution and may average over mechanically distinct compartments, whereas AFM captures micron-scale heterogeneity. All values reported as mean ± SD or ranges as published.

This controversy is not merely technical—it has profound implications for understanding GSC niche mechanics. If the perivascular niche (where GSCs preferentially reside) is mechanically distinct from the tumor core or invasive margin, then GSCs may experience a specific mechanical microenvironment that differs from the bulk tumor properties measured by MRE. To date, no study has systematically mapped the stiffness of known GSC niches at cellular resolution within human GBM tissue, representing a critical gap in the field.

### 2.3 Beyond stiffness: solid stress, fluid stress, and ECM topology

As understanding matured, the field recognized that stiffness represents only one component of a complex mechanical landscape. Jain et al. systematically categorized tumor mechanical forces into solid stress (from structural components), fluid stress (from vascular and interstitial flow), and matrix properties (stiffness and topology) (Jain et al., 2014). In brain tumors, solid stress is uniquely constrained by the rigid skull, such that tumor expansion generates compressive forces sufficient to cause neuronal loss and neurological dysfunction (Seano et al., 2019).

Kalli et al. provided direct evidence that compressive stress regulates brain cancer cell migration through MEK1/Erk1 pathway activation and GDF15 expression, demonstrating that the less aggressive H4 glioma cells showed stronger compression-induced migration enhancement than the highly aggressive A172 GBM cells (Kalli et al., 2019). This finding introduced an important nuance: the mechanical sensitivity of brain tumor cells is cell-type- and context-dependent, with less aggressive cells potentially more susceptible to mechanical transformation.

ECM topology—the three-dimensional architecture of the matrix—provides another critical layer of mechanical regulation. GBM cells characteristically migrate along blood vessels and white matter tracts, indicating that aligned topographical cues guide directional invasion (Cuddapah et al., 2014). This contact guidance phenomenon was demonstrated using electrospun nanofiber scaffolds that mimic white matter tract topography, showing that GBM cell migration speed depends on both fiber alignment and fiber stiffness (Rao et al., 2013a). Beyond the ECM network, the cellular architecture of the tumor itself imposes profound mechanical regulation, most notably through the formation of tumor microtubes (TMs). Pioneering *in vivo* imaging by Osswald and colleagues revealed that glioma cells, particularly those exhibiting stem-like properties, extend ultralong, F-actin-rich membrane processes (TMs) to form a complex, interconnected syncytium (Osswald et al., 2015). Mechanobiologically, this TM network is not merely a conduit for biochemical communication, but a biomechanically resilient scaffold that allows GSCs to physically resist intense solid stress and surgical mechanical damage (Huang et al., 2025; Lin et al., 2024). Furthermore, fluid dynamics actively orchestrate GSC behavior. Interstitial fluid pressure (IFP) and the resulting fluid shear stress are significantly elevated in GBM. Kingsmore et al. demonstrated that elevated interstitial fluid flow directly increases patient-derived GSC invasion through CD44-mediated mechanotransduction mechanisms (Kingsmore et al., 2016). Thus, solid stress, fluid dynamics, and TM-mediated mechanical resilience form an integrated biophysical shield that protects GSCs.

### 2.4 Mechanical heterogeneity of distinct GSC niches

To accurately understand mechanotransduction in GSCs, it is imperative to dissect the specific biophysical properties of the distinct anatomical niches where GSCs reside (Hambardzumyan and Bergers, 2015):

1. The Perivascular Niche (PVN): The PVN is a comparatively rigid compartment within the soft brain. Due to the high local concentration of basement membrane proteins (laminins, collagen IV) and continuous cyclic mechanical strain from pulsatile blood flow, this high-stiffness microenvironment robustly activates integrin/FAK and Notch signaling, which are essential for maintaining the aggressive GSC pool (Lathia et al., 2015).

2. The Hypoxic/Peri-necrotic Niche: Characterized by collapsed vasculature and rapid cellular turnover, this core region presents a unique mechanical paradox: it exhibits lower overall ECM stiffness but exerts extreme compressive solid stress. This severe compression forces GSCs to undergo HIF-dependent metabolic reprogramming and transition into a more quiescent, drug-resistant state (Huang et al., 2025; Bi et al., 2024).

3. The Invasive Margin (White Matter Tracts): At the tumor boundary, GSCs encounter highly aligned bundles of myelinated axons. Here, mechanical topography dominates over absolute stiffness. The aligned hyaluronic acid and myelin tracts provide physical “contact guidance,” activating Rho/ROCK and Piezo1 pathways to drive rapid, highly directional single-cell invasion into the healthy parenchyma (Cuddapah et al., 2014; Rao et al., 2013a).

## 3 Mechanosensing and mechanotransduction: molecular mechanisms in glioma

### 3.1 The integrin-focal adhesion-cytoskeleton axis

The molecular machinery through which glioma cells sense and respond to mechanical cues has been elucidated through studies converging from two research traditions: the broader mechanobiology field and glioma-specific investigations. Integrins, the primary cell-ECM adhesion receptors, undergo force-dependent conformational changes that initiate mechanotransduction cascades (Zhou et al., 2022).

In the glioma context, Ulrich et al. established that glioma cells are mechanosensitive, showing that multiple established bulk GBM cell lines (U373-MG, U87-MG, U251-MG, SNB19, C6) exhibited increased spreading, migration speed, and proliferation on stiffer substrates, and that this sensitivity required non-muscle myosin II and ROCK activity (Ulrich et al., 2009). However, a paradoxical finding by Wong et al. revealed that patient-derived glioma tumor-initiating cells (L0 and L2), unlike established cell lines, did not show sensitivity to substrate stiffness unless MLCK or ROCK was constitutively activated (Wong et al., 2015). This observation—that GSCs may be mechanically “insensitive” in their native state—is deeply significant, as it raises the possibility that the acquisition of mechanosensitivity itself may represent a transition in GSC fate, a concept that requires further direct validation.

Sen et al. demonstrated that α-actinin isoforms make isoform-specific contributions to glioma cell mechanobiology, linking cytoskeletal architecture to ECM mechanosensing (Sen et al., 2009). Umesh et al. further showed that ECM stiffness enhances glioma cell proliferation by stimulating EGFR signaling, and that inhibition of EGFR reduced stiffness-sensitivity (Umesh et al., 2014).

### 3.2 CD44-hyaluronic acid: a brain-specific mechanotransduction axis

A distinguishing feature of glioma mechanobiology is the central role of CD44-HA interactions, reflecting the HA-rich composition of brain ECM. Kim and Kumar demonstrated that CD44-mediated adhesion to HA contributes to mechanosensing and invasive motility in glioma (Kim and Kumar, 2014). Rape et al. developed a composite hydrogel system showing that matrix-bound HA-CD44 interactions reduce migration speed at tissue interfaces, indicating that HA serves as a mechanical “brake” on glioma cell movement (Rape and Kumar, 2014).

The downstream consequences of HA-CD44 signaling for GSC fate are substantial. CD44 activates PI3K/AKT, RhoGTPases, and Hippo signaling pathways (Xu et al., 2010). Xiao et al. demonstrated that HA concentration in the matrix, combined with ECM stiffness, determines drug resistance in patient-derived GBM cells through CD44-dependent upregulation of p-AKT (Xiao et al., 2018). This work established that mechanical and biochemical cues act cooperatively—not independently—to determine GSC therapeutic responsiveness.

### 3.3 The Hippo-YAP/TAZ pathway as a mechanical integrator

The Hippo-YAP/TAZ pathway has emerged as a central integrator of mechanical signals in glioma. When the upstream Hippo kinase cascade is inactive—as occurs under high cytoskeletal tension on stiff substrates—unphosphorylated YAP/TAZ translocates to the nucleus and activates TEAD-mediated transcription of pro-proliferative and pro-invasive genes (Dupont et al., 2011). Elosegui-Artola et al. elucidated the molecular clutch mechanism by which matrix rigidity controls force transmission and YAP nuclear entry through a talin-vinculin-integrin-fibronectin clutch (Elosegui-Artola et al., 2016).

In glioma, YAP-dependent mechanotransduction has been shown to be required for proliferation and migration on native-like substrate topography (Mascharak et al., 2017). However, the specific role of YAP/TAZ in GSC self-renewal versus differentiation decisions under different mechanical conditions remains poorly defined.

### 3.4 Piezo1: an emerging mechanosensitive ion channel in glioma

Chen et al. identified a feedforward mechanism in which the mechanosensitive ion channel Piezo1 senses tissue mechanics and promotes glioma aggression (Chen et al., 2018). Further mechanistic investigations have revealed that Piezo1 physically localises to the focal adhesions of glioma cells, catalysing their maturation and growth through a force-dependent calcium signalling pathway (Yao et al., 2022). Rather than operating independently, this direct mechano-electrical transduction pathway is deeply integrated with the tumour microenvironment; Piezo1 activation is closely associated with extracellular matrix (ECM) interactions, actin cytoskeleton remodelling, and the activation of integrin adhesion signalling (Chen et al., 2018). By transducing mechanical stimuli –such as the pressure gradients generated by the tumour itself –Piezo1 facilitates a mechanosensory advantage that promotes tumourigenesis and correlates with higher malignancy and poor clinical prognosis (Chen et al., 2018; Pogoda et al., 2014; Qu et al., 2020a; Qu et al., 2020b). However, whether Piezo1 plays a role specifically in the fate determination of patient-derived GSCs --as opposed to the behaviour of bulk tumour cells --remains an open question that warrants targeted investigation.

**FIGURE 3:**
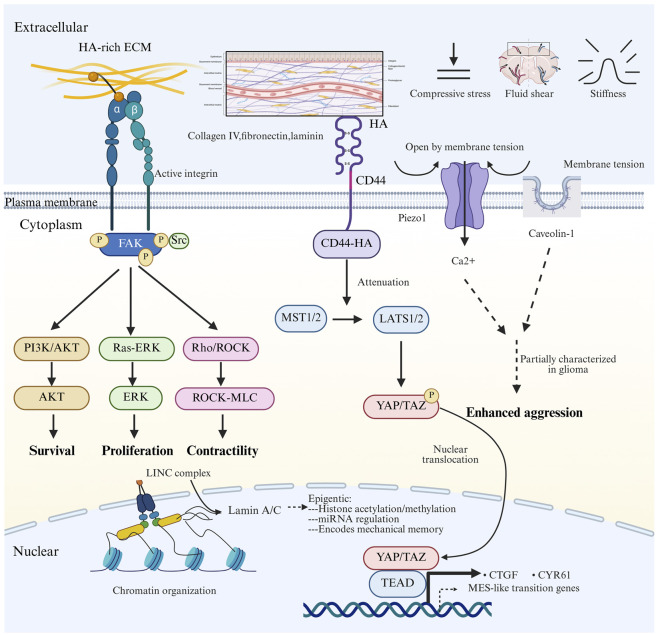
Hierarchical mechanosensing and mechanotransduction networks regulating glioma and glioma stem cell (GSC) plasticity. Extracellular mechanical cues—including matrix stiffness, compressive stress, and fluid shear—are perceived by distinct membrane-associated mechanosensors, primarily integrins, CD44, Piezo1, and caveolins. These sensors transduce physical forces into parallel intracellular biochemical cascades. The integrin-FAK axis regulates survival, proliferation, and contractility via PI3K/AKT, Ras-ERK, and Rho/ROCK pathways, respectively. The CD44-HA axis attenuates the Hippo kinase cascade (MST1/2-LATS1/2), facilitating YAP/TAZ nuclear translocation. Concurrently, mechanical signals are physically propagated to the nucleus via the LINC complex, mechanically coupling cytoskeletal tension to the nuclear lamina (Lamin A/C). Note on evidence levels: To rigorously distinguish validated mechanisms from emerging hypotheses, solid arrows are exclusively used to represent fully established signaling pathways with direct experimental evidence in patient-derived GSCs or established bulk glioma models. Dashed arrows denote hypothetical pathways, indirect mechanisms, or emerging concepts—specifically, Piezo1/Caveolin-1 downstream cascades, LINC-mediated mechano-epigenetics (encoding mechanical memory), and YAP/TAZ/TEAD-driven mesenchymal (MES)-like state transitions. These dashed mechanisms raise the possibility of novel biophysical regulations but are currently extrapolated from other solid tumors or general mechanobiology, and thus require direct experimental validation in primary GSC models.Created in BioRender. Ai, Y. (2026) https://BioRender.com/hf9s7x0.

The mechanosensing and mechanotransduction pathways discussed in Sections 3.1–3.4 are integrated into a comprehensive molecular pathway diagram in Figure 3, which illustrates the hierarchical organization of mechanotransduction from extracellular mechanical inputs to nuclear transcriptional outputs in GSCs. As depicted, extracellular mechanical cues—including matrix stiffness (ranging from 0.1 to 1 kPa in normal brain to 0.07–13.5 kPa in GBM (Miroshnikova et al., 2016)), compressive solid stress, and fluid shear forces—are sensed by four classes of membrane-associated mechanosensors: integrins engaging collagen IV, fibronectin, and laminin of the vascular basement membrane; CD44 receptors binding hyaluronic acid in the brain-specific ECM; Piezo1 mechanosensitive ion channels activated by membrane tension (Chen et al., 2018); and caveolins responding to membrane flattening. These sensors activate three major intracellular signaling cascades that operate in parallel but with extensive crosstalk. The integrin-FAK axis (Pathway 1) proceeds through FAK autophosphorylation, Src recruitment, and bifurcation into PI3K/AKT (survival), Ras-ERK (proliferation), and Rho/ROCK-MLC (contractility) branches (Ulrich et al., 2009; Umesh et al., 2014). The CD44-Hippo/YAP axis (Pathway 2) operates through attenuation of the MST1/2-LATS1/2 kinase cascade, permitting unphosphorylated YAP/TAZ to translocate to the nucleus and activate TEAD-dependent transcription of stemness-associated genes including CTGF and CYR61 (Kim and Kumar, 2014; Dupont et al., 2011). The Piezo1-Ca^2+^ axis (Pathway 3) involves force-induced channel opening and calcium influx, with downstream signaling cascades that remain partially characterized in the glioma context (Chen et al., 2018). Critically, as shown in the nuclear compartment of Figure 3, mechanical signals are transmitted to the nucleus through the LINC complex (Nesprin-SUN proteins) connecting the cytoskeleton to the nuclear lamina (Broders-Bondon et al., 2018), where they can alter chromatin organization through lamin A/C-dependent mechanisms and encode persistent epigenetic modifications—including histone acetylation/methylation changes and non-coding RNA expression—that may constitute the molecular basis of mechanical memory (Cambria et al., 2024). This integrated pathway architecture highlights the remarkable diversity of mechanosensing mechanisms available to GSCs and raises the important question of how pathway selection and prioritization are determined in different mechanical niches.

## 4 Mechanical regulation of GSC-specific behaviors

### 4.1 The 2D versus 3D stiffness paradox

A critical and underappreciated challenge in translating mechanobiological findings to GSC biology is the striking discrepancy between cell responses on 2D substrates versus within 3D matrices. On 2D polyacrylamide hydrogels, glioma cells consistently show increased proliferation, spreading, and migration with increasing stiffness (Umesh et al., 2014; Ulrich et al., 2010). Crucially, this paradox is strongly influenced by the biological model employed. Established cell lines (e.g., U87-MG) have been cultured for decades in serum on rigid polystyrene (∼3 GPa), leading to an artificially selected, “stiffness-addicted” integrin repertoire. Consequently, they often exhibit enhanced proliferation in stiffer 3D gels{Lopardo, 2025 #1149}. Conversely, *bona fide* patient-derived GSCs, which are typically cultured in serum-free neurospheres, retain their evolutionary preference for the ultra-soft, HA-rich environment of the native brain. When encapsulated in 3D, GSCs thrive in softer regimes (e.g., 40–300 Pa). Furthermore, from a biophysical perspective, the paradox stems from the fundamental limitations of natural hydrogels. In 3D hydrogel systems, the relationship is often inverted: GBM cell spheroids display higher invasion in softer matrices (Ulrich et al., 2010). Patient-derived GBM cells showed robust growth in soft PEG hydrogels (40 Pa) but not in stiff ones (1000 Pa), while immortalized U87 cells exhibited the opposite pattern (Wang et al., 2020).

This paradox has important implications for GSC biology. In 3D, increasing matrix stiffness is typically achieved by increasing polymer concentration, which simultaneously reduces pore size and alters ligand clustering. As highlighted by Kondapaneni et al., “decoupling of matrix stiffness and porosity is typically challenging in 3D cultures” (Kondapaneni et al., 2024). Therefore, diminished GSC invasion in stiff 3D hydrogels may not indicate a lack of mechanosensitivity, but rather that the physical barrier (steric hindrance) outweighs the pro-migratory signals from increased stiffness. The field needs experimental systems that can independently vary stiffness, porosity, and ligand density to resolve this paradox in the GSC context.

**FIGURE 4:**
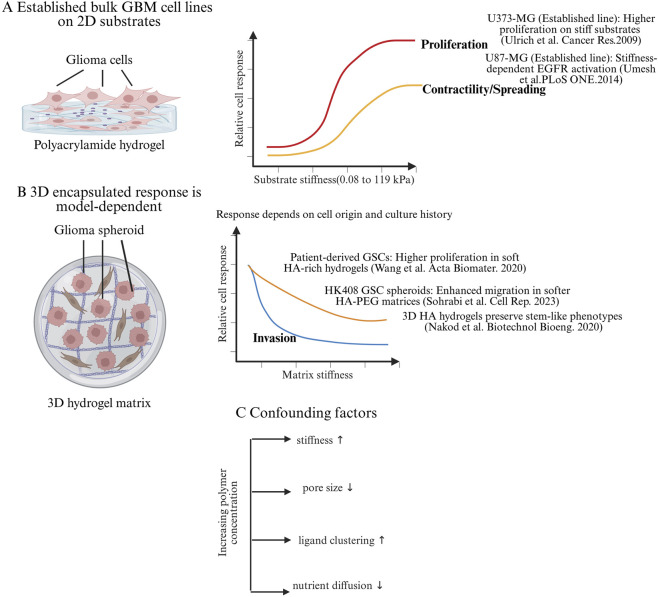
The 2D versus 3D stiffness response paradox and the influence of cell identity. **(A)** On 2D substrates, established bulk glioma cell lines (e.g., U373-MG, U87-MG) typically exhibit a monotonic positive relationship between substrate stiffness and malignant behaviors, showing increased proliferation, migration, and spreading on stiffer matrices. **(B)** In 3D encapsulated cultures, this relationship is frequently inverted. Notably, patient-derived primary GBM cells and *bona fide* glioma stem cells (GSCs, e.g., HK408) generally demonstrate preferential invasion and proliferation in softer matrices, highlighting a divergence from responses observed in 2D or with some established lines. **(C)** This 2D-3D paradox may be attributed to two main categories of confounding factors. First, biophysical constraints in 3D systems: increasing polymer concentration to elevate stiffness simultaneously decreases pore size and restricts nutrient diffusion, while also altering HA concentration and ligand density in natural hydrogels. Second, biological identity: established serum-cultured cell lines and patient-derived GSCs likely possess distinct intrinsic mechanosensitivities. Resolving this paradox in the GSC context requires advanced experimental platforms capable of decoupling macroscopic bulk stiffness from cellular-scale porosity and ligand presentation (References: Ulrich et al., 2009; Umesh et al., 2014; Ulrich et al., 2010; Wang et al., 2020; Sohrabi et al., 2023). Created in BioRender. Ai, Y. (2026) https://BioRender.com/ogt5kfu.

The discrepancy between glioma cell responses to stiffness in 2D versus 3D culture systems, and the confounding factors that underlie this paradox, are summarized schematically in Figure 4. As shown in Figure 4A, on 2D polyacrylamide substrates, glioma cells exhibit a monotonic positive relationship between substrate stiffness and cellular responses: U373-MG cells demonstrate significantly increased proliferation and migration speed on stiff (119 kPa) compared with soft (0.08 kPa) substrates (Ulrich et al., 2009), and U87-MG cells show enhanced EGFR phosphorylation with increasing stiffness (Umesh et al., 2014). In stark contrast, as illustrated in Figure 4B, when glioma cells are encapsulated within 3D hydrogel matrices, the relationship is frequently inverted: U373-MG spheroids exhibit higher invasion rates in soft (4–12 Pa) compared with stiff (∼1 kPa) collagen-agarose gels (Ulrich et al., 2010); patient-derived GBM cells show robust proliferation in soft PEG hydrogels (40 Pa) but minimal growth in stiff hydrogels (1000 Pa) (Wang et al., 2021); and HK408 GSC spheroids migrate more extensively in soft HA-PEG hydrogels (0.34 kPa) than in stiff ones (3.8 kPa) (Sohrabi et al., 2023). Figure 4C highlights the critical confounding factors that complicate interpretation of 3D results: in most hydrogel systems, increasing polymer concentration to achieve higher stiffness simultaneously decreases pore size, increases ligand density (in natural hydrogels), and reduces nutrient diffusion, making it impossible to attribute cellular responses solely to stiffness. This 2D-3D paradox is of particular relevance to GSC biology because GSCs reside within 3D niches *in vivo*, yet much of our mechanistic understanding of glioma mechanosensitivity derives from 2D studies. Resolving this paradox will require next-generation biomaterial platforms—such as interpenetrating network hydrogels or photo-tunable systems—that enable independent modulation of mechanical and transport properties within GSC-relevant 3D environments.

### 4.2 Mechanical regulation of dynamic GSC state plasticity

The conceptualization of GSCs has evolved dramatically. Rather than a static apex of a unidirectional differentiation hierarchy, recent single-cell transcriptomic milestones by Neftel, Suva, and colleagues have established that GBM comprises highly plastic cells dynamically transitioning among four primary states: neural-progenitor-like (NPC-like), oligodendrocyte-progenitor-like (OPC-like), astrocyte-like (AC-like), and mesenchymal-like (MES-like) (Neftel et al., 2019). Recognizing this plasticity fundamentally reshapes our interpretation of mechanobiology: mechanical cues do not simply turn static stemness “on” or “off”; rather, they act as profound biophysical stressors that may force transcriptomic state transitions.

While biochemical and immune drivers of these transitions are documented (Hara et al., 2021), the role of niche mechanics is an emerging hypothesis. It is postulated that extreme mechanical conditions—such as the high ECM stiffness and elevated solid stress found in fibrotic regions—may preferentially drive glioblastoma cells toward the therapy-resistant MES-like state. Conversely, softer, HA-rich environments that mimic the native brain parenchyma preferentially support the maintenance of OPC-like or NPC-like states. For instance, Nakod et al. demonstrated that 3D biomimetic HA hydrogels optimally preserve specific GSC phenotypes in patient-derived cells (Nakod et al., 2020), suggesting that matching native mechanical impedance prevents artifactual state transitions *in vitro*. Consequently, “mechanical differentiation” in GBM should be cautiously redefined as the potential force-dependent reprogramming of cells into distinct, environmentally adapted states.

### 4.3 Mechanically regulated drug resistance

The mechanical TME plays a demonstrably important role in GBM therapeutic resistance. The evidence has accumulated progressively: Erickson et al. showed stiffness-dependent temozolomide resistance in 3D chitosan-HA scaffolds (ED50 = 255 μM on soft 1.41 kPa scaffolds versus 3840 μM on stiff 27.7 kPa scaffolds) (Erickson et al., 2018). Wang et al. confirmed this stiffness-resistance relationship in patient-derived GBM cells using PEG hydrogels (Wang et al., 2021).

Importantly, Xiao et al. showed that ECM composition acts synergistically with stiffness: GBM cells in hydrogels with high HA concentration attained drug resistance more rapidly on softer matrices (by day 9 at 1 kPa vs. day 12 at 2 kPa), demonstrating that compositional cues can override stiffness effects under certain conditions (Xiao et al., 2018). Pedron et al. further established that HA composition influences erlotinib resistance independently through CD44-mediated p-STAT3 activation (Pedron et al., 2019).

The comprehensive body of evidence on how ECM stiffness and composition influence specific GBM cell behaviors is systematically organized in Table 2, which highlights the culture configuration (2D versus 3D), hydrogel platform, cell type (established lines versus patient-derived cells), and key mechanistic pathways implicated. As this tabulation reveals, several important patterns emerge. First, on 2D substrates, increasing stiffness consistently promotes proliferation, migration, and spreading across multiple cell lines and ECM ligands (Ulrich et al., 2009; Umesh et al., 2014). Second, in 3D systems, the stiffness-response relationship is cell-type-dependent, with patient-derived GSCs often showing preferential growth and migration in softer matrices (Sohrabi et al., 2023; Wang et al., 2021), while established cell lines such as U87 may respond differently (Rao et al., 2013a). Third, ECM composition—particularly HA concentration—exerts effects that can be independent of, additive to, or even dominant over stiffness effects on drug resistance and invasion (Xiao et al., 2018; Pedron et al., 2017). Fourth, drug resistance is consistently enhanced at higher stiffness in 3D scaffolds for bulk GBM cells (Wang et al., 2021; Erickson et al., 2018), but the interaction with HA can modulate this relationship in complex, non-linear ways (Xiao et al., 2018). These observations collectively argue that the mechanical regulation of GBM cell behavior cannot be reduced to a single parameter (stiffness) but must account for the combinatorial interplay of stiffness, composition, dimensionality, and cell identity.

**TABLE 2:** Effects of ECM stiffness and composition on GBM cell behaviors in tissue-mimetic models.

Cell line/Type	Culture config.	Biomaterial	Stiffness range	Key observation	Pathway/Mechanism	References
Migration/invasion						
U373-MG, U87-MG, U251-MG, SNB19, C6 (established bulk lines)	2D top-seeded	Polyacrylamide + fibronectin	0.08–119 kPa	Migration speed increases with stiffness	Non-muscle myosin II and ROCK dependent	Ulrich et al. (2009)
U373-MG spheroids (established bulk lines)	3D encapsulated	Collagen-agarose	4 Pa–1 kPa	Higher invasion in softer hydrogels	Steric barrier at high agarose concentration	Ulrich et al. (2010)
Patient-derived OSU-2(patient-derived GSCs)	3D encapsulated	Collagen I/III + HA	0.3–2.1 kPa	Migration decreases with increasing HA and stiffness	HA-CD44 interactions	Rao et al. (2013b)
HK177, HK408 spheroids (patient-derived GSCs)	3D encapsulated	HA-PEG + RGD	0.34 and 3.8 kPa	Soft hydrogels enhance migration and aerobic glycolysis	Metabolic reprogramming	Sohrabi et al. (2023)
Patient-derived OSU-2 (patient-derived GSCs)	2D top-seeded	Core-shell nanofibers	2.4–33.3 MPa	Fastest migration at intermediate modulus (PCL, 7.9 MPa)	Topography-stiffness interaction	Rao et al. (2013a)
GSC neurospheres (patient-derived GSCs)	2D top-seeded	Polyacrylonitrile nanofibers	3–1260 kPa	Optimal migration at 166 kPa	MGAT5-dependent mechanosensing	Marhuenda et al. (2021)
Proliferation						
U373-MG, U87-MG (established bulk lines)	2D top-seeded	Polyacrylamide + fibronectin	0.08–119 kPa	Proliferation increases with stiffness	EGFR pathway activation	Umesh et al. (2014)
U87-MG (established bulk lines)	3D encapsulated	PEG + MMP-cleavable + RGD	1–26 kPa	Soft hydrogels (1 kPa) enhance proliferation	—	Wang et al. (2014)
Patient-derived (aGBM, pGBM, DIPG) (patient-derived GSCs)	3D encapsulated	PEG + RGD	0.04 and 1 kPa	Patient-derived cells: robust growth in soft gels; U87: growth in stiff gels	Cell-type dependent response	Wang et al. (2020)
D-270 MG (patient-derived PDX cells)	3D gradient	PEG + MMP-cleavable + RGD	40–1300 Pa	Higher proliferation and MMP expression in soft zones	MMP-1, MMP-2 upregulation	Zhu et al. (2021)
Drug resistance						
U87-MG (established bulk lines)	2D top-seeded	Chitosan-HA	1.41–27.7 kPa	Stiff scaffolds increase TMZ ED50 (255→3840 μM)	ABCG2, CD44, MMP-2 upregulation	Erickson et al. (2018)
D-270 MG (patient-derived)	3D encapsulated	PEG + RGD + MMP-cleavable	0.04–26.6 kPa	Stiffness increases TMZ resistance	—	Wang et al. (2021)
GBM39, HK301, HK423(Patient-derived GSCs)	3D encapsulated	HA-PEG + RGD	1 and 2 kPa	High HA (0.5%) + stiffness → erlotinib resistance	CD44-p-AKT activation; faster resistance on soft (1 kPa)	Xiao et al. (2018)
GBM6, GBM12(Patient-derived PDX cells)	3D encapsulated	GelMA + HA	15.3 and 17.5 kPa	HA composition independently influences erlotinib response	EGFR-CD44-p-STAT3	Pedron et al. (2019)
HK301, GBM6, GS024, GS025 (patient-derived GSCs)	3D encapsulated	HA-PEG + RGD	1 kPa	High HA + RGD → TMZ/carmustine resistance	CD44 + integrin-αv → Src → BCL-2 suppression	Xiao et al. (2020)

Summary of studies examining the effects of ECM, stiffness and composition on GBM, cell migration, proliferation, and drug resistance. Note the consistent trend: on 2D substrates, stiffness promotes proliferation and migration, whereas in 3D, softer matrices often favor invasion and growth of patient-derived cells. Drug resistance generally increases with stiffness and HA, content. TMZ, temozolomide. All stiffness values as reported in original publications.

These findings collectively argue that drug resistance in GBM cannot be understood solely through genetic or biochemical analyses—the mechanical and compositional context must be considered. However, whether these resistance mechanisms are specific to the GSC subpopulation, or represent a bulk tumor cell response, has not been clearly delineated.

### 4.4 Mechano-metabolic coupling in GSC plasticity

An exciting frontier bridging biophysics and GSC state plasticity is mechano-metabolic coupling—defined here as the process by which extracellular physical forces are transduced into intracellular signals that directly alter cellular metabolic flux and nutrient utilization. As noted earlier, Sohrabi et al. recently provided the first direct evidence in GBM that microenvironmental stiffness induces metabolic reprogramming toward aerobic glycolysis (Sohrabi et al., 2023). However, relying on a single context is insufficient; insights from broader solid tumor mechanobiology offer a robust roadmap for future GSC research.

In breast and stromal cancers, it has been elegantly established that cytoskeletal tension and YAP/TAZ activation directly upregulate glycolytic enzymes and glutaminolysis to meet the high energetic demands of stiff-matrix-induced proliferation (Enzo et al., 2015). Furthermore, recent discoveries highlight that ECM stiffness fundamentally dictates mitochondrial dynamics; high mechanical forces restrict mitochondrial fission, thereby shifting the balance of ATP production from oxidative phosphorylation to glycolysis (Romani et al., 2019). Applied to the dynamic GSC model, we hypothesize that as GSCs transition into the highly contractile MES-like state in response to stiff fibrotic niches, they may undergo a forced mechano-metabolic shift to fuel enhanced invasion and therapy resistance. Deciphering how distinct mechanical niches spatially organize the metabolic dependencies of different GSC states presents a critical, unexplored therapeutic vulnerability that requires rigorous validation in primary GSC models.

## 5 Emerging concepts: mechanical memory and biophysical evolution

An exciting frontier in cancer mechanobiology—with particular relevance to GSC biology—is the concept of mechanical memory—defined as the ability of cells to retain phenotypical adaptations and signaling states induced by a past mechanical environment, even after migrating into a new physical niche. Balestrini et al. first described this phenomenon in fibroblasts, showing that cells primed on stiff substrates retained myofibroblast phenotypes even after transfer to soft substrates (Balestrini et al., 2012). Cambria et al. recently proposed that mechanical memory, encoded through mechano-epigenetics (the stable alteration of chromatin architecture and DNA/histone modifications by physical forces). Persistent epigenetic changes including histone modifications and non-coding RNAs, enables tumor cells to retain biophysical adaptations acquired in the primary TME throughout the metastatic process (Cambria et al., 2024).

Watson et al. provided direct evidence of this concept in breast cancer, demonstrating that stiffness-induced RUNX2 activation and preserved chromatin accessibility instruct osteolytic bone metastasis (Watson et al., 2021). While gliomas rarely metastasize outside the brain, extrapolating this concept to GBM raises the possibility that GSCs originating in mechanically distinct niches (e.g., stiff perivascular zones vs. soft tumor core) might retain niche-specific epigenetic programming as they invade through the brain parenchyma, potentially explaining the phenotypic heterogeneity observed in recurrent tumors.

Broders-Bondon et al. further emphasized that mechanotransduction in tumor progression may represent a pathological reactivation of developmental mechanosensitive pathways, with the β-catenin pathway and YAP/TAZ serving as key mediators (Broders-Bondon et al., 2018). Whether mechanical memory contributes to the extraordinary treatment resistance and inevitable recurrence of GBM through GSC-specific epigenetic reprogramming represents a largely unexplored but potentially transformative research direction.

## 6 Experimental platforms: progress and limitations

### 6.1 From simple substrates to brain-mimetic systems

The experimental platforms for studying glioma mechanobiology have evolved substantially. Early studies relied on polyacrylamide hydrogels coated with ECM proteins (Ulrich et al., 2009), which, while providing tunable stiffness, fail to recapitulate the 3D, HA-rich environment of brain tissue. The development of HA-based hydrogels (Ananthanarayanan et al., 2011) and composite collagen-HA systems (Rao et al., 2013b) represented important advances toward brain-relevant substrates.

PEG-based synthetic hydrogels with independently tunable stiffness, degradability, and biochemical cues have enabled more controlled investigations (Wang et al., 2014). Gradient hydrogels with spatially varying stiffness within a single construct have provided insights into mechanotactic responses (Zhu et al., 2021).

### 6.2 Microfluidic and organ-on-chip integration

The integration of microfluidics with tunable matrix mechanics has enabled the study of glioma cell behavior under combined biochemical and mechanical gradients (Dou et al., 2020). Perivascular niche-on-chip devices have been shown to maintain the stemness of patient-derived GSCs in 3D environments (Gerigk et al., 2021). Critically, microfluidic migration assays have demonstrated clinical predictive value: Wong et al. showed that high GBM cell motility in microfluidic devices correlated with poor patient prognosis (Wong et al., 2021).

Despite these advances, current *in vitro* systems still fail to simultaneously recapitulate the key features of the GSC niche: soft, HA-rich ECM with perivascular architecture, heterogeneous cell populations (including endothelial cells, pericytes, and immune cells), and the spatial gradients of stiffness, oxygen, and nutrients that characterize the *in vivo* tumor.

To synthesize the complex and often context-dependent mechanobiological findings discussed throughout this review, we provide a comprehensive summary in Table 3. This table delineates the essential components of the “mechano-stemness axis”—linking specific extracellular mechanical cues to their primary mechanosensors, downstream signaling cascades, and ultimate phenotypic outputs. Crucially, addressing the heterogeneity in current literature, the table explicitly distinguishes mechanisms validated in *bona fide* patient-derived GSCs from those observed in established bulk GBM cell lines, and highlights the corresponding therapeutic implications for future mechanotherapeutics.

**TABLE 3:** Core components of the proposed “mechano-stemness axis” in brain tumors.

Mechanical cues	Primary mechanosensors	Key downstream signaling	Phenotypic/Stemness output	Model system evidence	References
High ECM stiffness (Collagen/Fibrillar)	Integrins (αvβ3, α5β1)	FAK, Rho/ROCK, MLC, YAP/TAZ nuclear translocation	Increased proliferation, contractility, potential MES-like transition	Established bulk lines (U87, U251); Extrapolated to GSCs	Dupont et al. (2011), Ulrich et al. (2009)
HA-rich soft matrix (∼40-300 Pa)	CD44	PI3K/AKT, Attenuated Hippo (Cytoplasmic YAP/TAZ)	Preserves OPC/NPC-like states, maintains slow-cycling stemness	Patient-derived GSCs (e.g., OSU-2, GBM39)	Kim and Kumar (2014), Nakod et al. (2020)
Interstitial Fluid Flow/ Fluid Shear Stress	CD44, Glycocalyx	CXCR4/CXCL12 axis, Integrin clustering	Promotes directional invasion along perivascular spaces	Patient-derived GSCs	Kingsmore et al. (2016)
Compressive Solid Stress	Caveolins, Piezo1	HIF-1α, MEK/ERK, GDF15	Induces quiescence, neuronal loss, enhances survival	Mouse orthotopic models; Bulk GBM cells	Seano et al. (2019), Kalli et al. (2019)
Aligned Topography (White Matter Tracts)	Integrins, CD44	Myosin II, Rho GTPases	Contact-guided, rapid single-cell invasion into parenchyma	Bulk cell lines & Patient-derived GSCs	Cuddapah et al. (2014), Rao et al. (2013a)

## 7 Critical gaps and future directions

Synthesizing the evidence reviewed above, several critical gaps emerge that constrain our understanding of mechanotransduction in GSC fate determination:

First, the mechanical properties of specific GSC niches remain uncharacterized. While bulk tumor stiffness has been measured by multiple techniques, no study has systematically mapped the stiffness of identified GSC niches—including the perivascular niche, the hypoxic core, and the invasive margin—at cellular resolution in human GBM specimens. The debate over whether GBM is stiffer or softer than normal brain may be resolved by recognizing that GSCs occupy mechanically distinct compartments within a heterogeneous tumor, but this hypothesis requires direct experimental validation.

Second, the mechanosensitivity of GSCs versus differentiated glioma cells is poorly understood. The finding by Wong et al. that GSCs are mechanically insensitive unless contractility is constitutively activated (Wong et al., 2015) suggests a fundamental difference in how GSCs process mechanical information. Whether this reflects a distinct mechanosensor repertoire, cytoskeletal organization, or nuclear mechanical properties of GSCs is unknown.

Third, the role of mechanical memory in GSC fate plasticity has not been investigated. Given that GSCs transition between different mechanical niches during invasion and that mechanical memory can encode persistent epigenetic changes (Cambria et al., 2024), understanding whether mechanical priming in the primary niche influences GSC behavior at recurrence sites is a critical question with therapeutic implications.

Fourth, the interplay between mechanical cues and metabolic reprogramming in GSCs is largely unexplored. The recent finding by Sohrabi et al. that microenvironmental stiffness induces metabolic reprogramming in glioblastoma (Sohrabi et al., 2023) opens a new dimension connecting mechanobiology with cancer metabolism, but the GSC-specific implications remain to be determined.

Fifth, there is a critical absence of integrated *in vitro* platforms that simultaneously recapitulate the mechanical, compositional, and cellular complexity of the GSC niche. Current systems address individual aspects but fail to capture the coupled multi-physics environment that GSCs experience *in vivo*.

Addressing these gaps requires a convergent approach that combines: (i) high-resolution mechanical characterization of patient GSC niches using correlative AFM and spatial transcriptomics; (ii) engineered 3D platforms that independently modulate stiffness, composition, and topology while supporting GSC culture; (iii) temporal tracking of mechanically induced epigenetic changes to assess mechanical memory; and (iv) integration of mechanical measurements with metabolic profiling to understand mechano-metabolic coupling. Such an approach has the potential to reveal new mechanotransductive determinants of GSC fate and identify novel therapeutic targets for this devastating disease.

**FIGURE 5:**
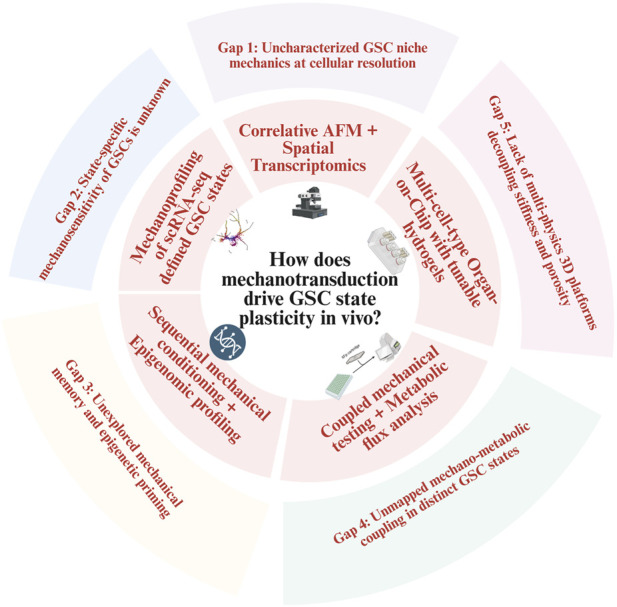
A convergent research framework for decoding mechanotransduction in dynamic GSC state plasticity. To answer the central question of how physical forces drive *in vivo* GSC state transitions, this framework identifies five interconnected knowledge gaps (outer ring) and pairs them with proposed state-of-the-art experimental solutions (inner ring). Gap 1 addresses the absence of cellular-resolution mechanical maps of native tumor niches, which can be resolved by integrating correlative AFM with spatial transcriptomics. Gap 2 challenges the static stemness paradigm, highlighting the need to mechanoprofile distinct, scRNA-seq-defined GSC states (e.g., OPC-like vs. MES-like) to reveal state-specific mechanosensitivities. Gap 3 points to the extrapolated and hypothesized concept of mechanical memory; validating whether GSCs retain niche-derived epigenetic priming requires sequential mechanical conditioning coupled with longitudinal epigenomic profiling. Gap 4 targets the unexplored mechano-metabolic coupling, proposing the integration of mechanical tuning with real-time metabolic flux analysis to determine how physical forces re-wire GSC energetics. Finally, Gap 5 addresses a critical biophysical bottleneck—the inability of conventional hydrogels to decouple bulk stiffness from porosity and ligand density. Overcoming this requires the engineering of multi-physics, multi-cell-type organ-on-chip platforms. Addressing these integrated gaps will ultimately translate *in vitro* mechanobiological observations into actionable, state-specific therapeutic vulnerabilities in glioblastoma. Created in BioRender. Ai, Y. (2026) https://BioRender.com/uc7gy8h.

The five critical research gaps identified above, together with the proposed experimental approaches to address them and the anticipated translational outcomes, are synthesized into a convergent research framework in Figure 5. As this framework illustrates, the central unresolved question—how mechanotransduction determines GSC fate in the native tumor niche—is obstructed by five interconnected knowledge gaps that span from tissue-level mechanics to molecular-level epigenetics. Gap 1 (uncharacterized GSC niche mechanics) can be addressed through correlative AFM and spatial transcriptomics applied to patient GBM specimens, which would for the first time map the mechanical properties of perivascular, hypoxic, and invasive GSC niches at cellular resolution and determine whether these compartments are mechanically distinct, as suggested by bulk tissue measurements (Miroshnikova et al., 2016; Sohrabi et al., 2023). Gap 2 (unknown GSC-specific mechanosensitivity) requires systematic comparative mechanobiology studies of prospectively sorted GSC and non-GSC populations from the same patient tumors, building on the intriguing observation by Wong et al. that GSCs exhibit mechanical insensitivity unless contractility is constitutively activated (Wong et al., 2015)—a finding that suggests GSCs may possess a fundamentally different mechanosensor repertoire or cytoskeletal organization than differentiated glioma cells. Gap 3 (unexplored mechanical memory in GSC fate plasticity) demands sequential mechanical conditioning experiments coupled with longitudinal epigenomic profiling (ATAC-seq, ChIP-seq, DNA methylation arrays) to determine whether GSCs that transit from stiff perivascular niches to soft parenchymal tissue retain niche-derived chromatin accessibility patterns, as has been demonstrated for breast cancer cells by Watson et al. (2021) and conceptualized more broadly by Cambria et al. (2024). Gap 4 (poorly understood mechano-metabolic coupling in GSCs) can be investigated through combined mechanical measurements and metabolic flux analysis (e.g., Seahorse assays, isotope tracing) on patient-derived GSCs cultured in mechanically defined environments, extending the recent demonstration by Sohrabi et al. that microenvironmental stiffness induces metabolic reprogramming toward aerobic glycolysis in GBM cells (Sohrabi et al., 2023) to the specific GSC subpopulation. Gap 5 (absence of integrated GSC niche-mimetic platforms) requires the development of multi-cell-type organ-on-chip devices incorporating tunable HA-based hydrogels, endothelial cells, pericytes, and immune cells—building on existing perivascular niche-on-chip models (Yi et al., 2019)—with independent control over stiffness, composition, oxygen tension, and interstitial flow. As indicated by the convergence arrows in Figure 5, addressing these gaps through an integrated, multidisciplinary approach will enable three translational advances: the identification of mechanotransduction-based therapeutic targets for GSC elimination or forced differentiation (e.g., Piezo1 inhibitors, YAP/TAZ modulators, FAK inhibitors); the development of non-invasive mechanical biomarkers through clinical MRE or SWE for monitoring GSC niche dynamics during treatment; and the establishment of personalized mechanical profiling paradigms in which patient-derived GSCs are characterized for their mechano-phenotype to guide individualized therapeutic strategies. This convergent framework positions mechanotransduction not as an ancillary feature of the GSC niche, but as a central and therapeutically actionable determinant of GSC fate that warrants dedicated investigation at the intersection of mechanobiology, stem cell biology, neuro-oncology, and bioengineering.

## 8 Conclusion

The body of evidence reviewed here establishes that the mechanical microenvironment of GBM is not a passive scaffold but an active, driving force of tumor cell behavior. The glioma mechanical landscape is profoundly heterogeneous (Table 1), with distinct tumor niches characterized by unique profiles of solid stress, fluid dynamics, and ECM stiffness values that span more than two orders of magnitude (Miroshnikova et al., 2016; Sohrabi et al., 2023). This complex biophysical heterogeneity is transduced through parallel mechanosensing pathways—such as integrin-FAK, CD44-HA, Piezo1, and YAP/TAZ (Figure 3)—into intracellular signaling cascades that orchestrate proliferation, migration, drug resistance, and crucially, dynamic GSC state plasticity (Table 2).

The highlighted 2D-3D stiffness paradox (Figure 4) underscores the critical necessity of decoupling macroscopic bulk stiffness from microscale porosity and ligand density, as well as the imperative to distinguish the artifactual mechanical responses of established, serum-cultured cell lines from the intrinsic mechanosensitivity of *bona fide* patient-derived GSCs. Furthermore, while currently extrapolated from other solid tumors and requiring direct validation in primary GSC models, emerging paradigms such as mechanical memory and mechano-metabolic coupling raise exciting possibilities for understanding how physical forces might program GSC identity over time and across space.

We propose that the “mechano-stemness axis” (Figure 1) represents a fundamental regulatory layer in GBM biology that operates in concert with, and cannot be separated from, the established biochemical signaling networks governing GSC state transitions. The convergent research framework outlined in Figure 5 provides a roadmap for systematically addressing the critical knowledge gaps that currently limit our ability to exploit mechanotransduction therapeutically. The clinical promise is substantial: unlike irreversible genetic mutations, the mechanical microenvironment is both pharmacologically and physically modifiable, offering an entirely new frontier of therapeutic strategies for the most lethal of brain tumors.
